# Parental Knowledge and Awareness about Shaken Baby Syndrome in Jeddah, Saudi Arabia: A Cross-Sectional Study

**DOI:** 10.3390/pediatric15020027

**Published:** 2023-05-05

**Authors:** Fatma Alzahrani, Basma A. Al-Jabri, Salah Aldeen L. Ramadan, Abdulaziz M. Alshehri, Abdulaziz S. Alsheikh, Hanan Hassan Mushaeb, Sara Faisal Albisher, Maab Sadek AlSwealh

**Affiliations:** 1 Pediatric Department, Faculty of Medicine, King Abdulaziz University Hospital, King Abdulaziz University, Jeddah 21589, Saudi Arabia; 2 Faculty of Medicine, King Abdulaziz University, Jeddah 21589, Saudi Arabia

**Keywords:** parental, knowledge, awareness, SBS, Jeddah, cross-sectional

## Abstract

Background: Despite the serious consequences of shaken baby syndrome (SBS), previous studies revealed a low level of knowledge among Saudi parents. Methods: This is a cross-sectional study. An electronic questionnaire was distributed through social media platforms to parents of pediatric age group children in Jeddah, Saudi Arabia. A total of 524 responses were received. Data about participants’ demographics, knowledge, attitude, and practice regarding SBS were collected through convenient random sampling. Results: A total of 524 responses were received; 30.7% of the participants were familiar with SBS. The Internet and the social media platforms were the most common sources of information. There was no statistically significant correlation between knowledge levels and participants’ sociodemographic factors; only 32.3% of individuals had good knowledge. Of them, 84% had a positive attitude towards learning more about SBS, and 40.1% and 34.3% were interested in learning more about SBS before and during pregnancy, respectively. Carrying the baby and shaking were the actions most frequently taken when a baby was crying. Of them, 23.9% forcefully shake their child, while 41.4% of them throw their infant up in the air and catch it. Conclusions: It is important to conduct health education programs on SBS for mothers throughout the prenatal period.

## 1. Introduction

The term shaken baby syndrome (SBS) is usually referred to the violent head trauma inflicted on infants and young children [1]. SBS was defined as “a devastating form of inflicted traumatic brain injury that occurs when a young child is violently shaken and subjected to rapid acceleration, deceleration and rotational forces, with or without impact” [2]. It was first described in 1984, when Ludwig and Warman examined 20 infants and young toddlers who had been shaken, and none of them had signs of a head impact injury [3]. Currently, The American Academy of Pediatrics (AAP) uses the term abuse head trauma (AHT) to indicate non-accidental brain damage resulting from impact trauma, shaking, or a combination of the two [4]. Internationally, the prevalence of SBS in infants less than one year was estimated to be 14–33.8/100,000 [5]. These numbers are allegedly lower than that and they are due to reporting issues [5,6]. In Saudi Arabia, there are no studies to report the accurate prevalence of SBS.

Significant symptoms of SBS include vomiting, irritability, breathing problems, lethargy, seizures, and coma [3]. Shaking-related injuries can cause death or severe neurological problems such as cerebral palsy, cortical blindness, static encephalopathy, mental retardation, and learning disabilities. In younger children under the age of one, subdural hemorrhages are more common [7]. Variable risk factors have been identified in relation to SBS/AH such as parental, environmental, and baby risk factors. Very low levels of education, substance or alcohol misuse, young moms, and unemployment are among parental high-risk variables, in addition to parents not expecting babies, single caregivers, and parental depression [5,6]. Another risk factor was postpartum depression, which was found to be an independent risk factor for shaking and smothering regardless of the perceived amount of crying [8,9]. Depressed mothers may have lower tolerance levels for infant crying [8,9,10]. Partner violence, home or social instability, and poverty are environmental risk factors for child abuse [11].

Ex-premature infants, those with disabilities or chronic illnesses, those who cry a lot, and those who are the consequence of an unplanned pregnancy are specific risk factors related to the infant [12,13]. It is established that the most frequent trigger for SBS/AHT is a crying infant [11]. Increasing the public awareness of SBS is crucial. This involves being aware of the risks of shaking a newborn erratically, as well as the causes, symptoms, risk factors, and preventative measures for SBS. Increasing public awareness could help fewer babies obtain SBS [14]. In Germany, Berthold had reported that 40% of participants had no prior knowledge of abusive head trauma (AHT) or shaken baby syndrome [15]. According to Foley (2013), he examined Turkish parents’ awareness of SBS, 50.3% were unaware that shaking a newborn can be harmful, while 24% believed that shaking would not be harmful [16]. Locally, a study conducted in Tabuk reported that 57.61% of the parents shake their babies to make them quite within the first year of life. More than half of the respondents 67.39% reported poor knowledge about the risks of shaking the baby during the first year of life [17]. In a similar study that was conducted in Riyadh, Alomran (2022), it was reported that the participants had poor knowledge and awareness about SBS, but, despite that, they expressed a positive attitude toward learning more about it [18]. The American Academy of Pediatrics recommends increased awareness and education programs for parents and caregivers about the dangers of shaking babies, as well as safe approaches to calm and cope with a crying infant, with the goal of prevention [10]. Shaken baby syndrome is a condition that can cause permanent neurological damage, resulting in enormous family and social costs. Having a high level of awareness about shaken baby syndrome, on the other hand, should help to prevent the development of its consequences [19]. To our knowledge, there was no study that tested the parental awareness about SBS in Jeddah. This study aims to evaluate the knowledge about SBS, and the sources of information among parents living in Jeddah city, Saudi Arabia.

### Subjects and Methods

Study design, setting, and time: a cross-sectional study was performed in Jeddah, Saudi Arabia from August to September 2022.

Study participants: the inclusion criteria were parents of children aged from birth to 14 years living in Jeddah, Saudi Arabia. Those who were living outside Jeddah were excluded from the study.

Sample size: the total number of children of 0–14 years in Jeddah was 739,146, with the use of a confidence interval of 95% and a margin of error of 5%, a total minimum sample of 384 parents was calculated using the Raosoft online calculator. The study included 524 participants.

Data collection: an electronic questionnaire was distributed on social media platforms to the target population using the convenient random sampling method and parents were asked to complete it. The questionnaire included data about participants’ demographics, knowledge, attitude, and practice regarding shaken baby syndrome (SBS). A knowledge score was computed based on summing up the correct responses to knowledge items (N = 8), where each correct answer was given 1 and incorrect answer was given 0. Therefore, the overall knowledge score ranged between 0 and 8. Good knowledge was defined as having a knowledge score above the median value.

Ethical considerations: an ethical approval was obtained from the research ethics committee of King Abdulaziz University, Jeddah, Saudi Arabia.

Statistical Analysis: Data analysis was carried out using RStudio (R version 4.1.1). We used frequencies and percentages to express categorical data, while numerical data were presented using median and interquartile range (IQR). Factors associated with the levels of knowledge and attitudes were assessed using a Pearson’s chi-squared test and a Fisher’s exact test. Predictors of positive attitudes were assessed by constructing a binary logistic regression analysis using the significantly associated variables from the inferential analysis as independent variables. The outcomes were expressed as odds ratio (OR) and 95% confidence intervals (95% CIs). A *p* value of <0.05 indicated statistical significance.

## 2. Results

### 2.1. Sociodemographic Characteristics

A total of 524 responses were received on the online platform. The majority of respondents were female (86.1%), Saudis (84.9%), and married (81.9%). The most common age categories were 30–39 years (29.0%) followed by 20–29 years (27.0%). Almost less than a half of the respondents were employees (49.6%) with a monthly income of <5000 SAR (43.1%), whereas more than a half of them had obtained a bachelor’s degree (60.1%). Notably, more than two-thirds of the sample had 1 to 5 children (69.8%). Anxiety and depression were prevalent among 11.3% and 3.8% of the respondents, respectively. Additionally, 10.7% of them had a child with a chronic illness (Table 1).

### 2.2. Description of Knowledge-Related Variables

**Figure 1:**
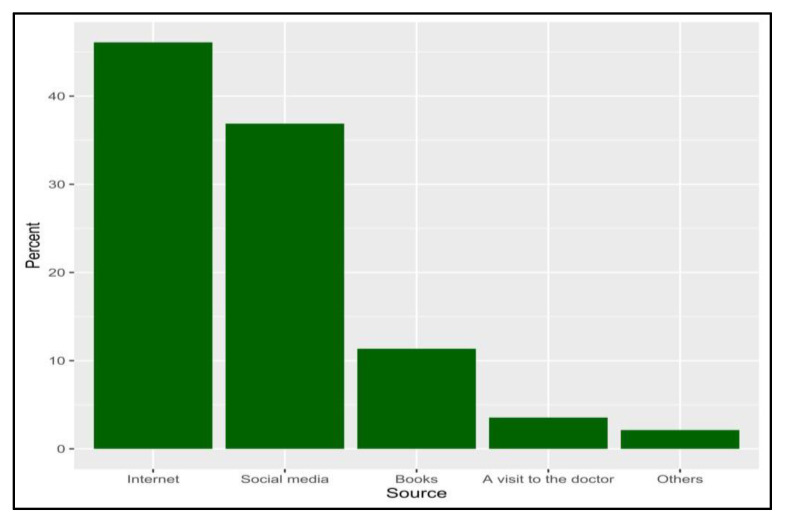
The percentage of sources that candidates obtain their knowledge about shaken baby syndrome from (N = 161).

Generally, 161 respondents (30.7%) declared that they knew about shaken baby syndrome (SBS). The most common sources of knowledge among knowledgeable participants were the Internet (46.1%) and social media (36.9%) (Figure 1). Focusing on the respondents who were knowledgeable about SBS (N = 161), the calculated knowledge score ranged between 0 and 8. The median (IQR) score was 4.0 (2.0 to 5.0), and 52 participants had a good knowledge level (32.3%). The association analysis showed no significant differences in knowledge levels based on the sociodemographic characteristics of the respondents (Table 2).

### 2.3. Attitudes towards Shaken Baby Syndrome

Out of the whole sample, the majority of participants expressed their interest to know about SBS (having a positive attitude, 84.0%), preferably via the Internet and social media (37.1%) and doctors/medical staff during the vaccination period (28.6%). Additionally, 40.1% and 34.3% of the respondents wanted to know SBS-related information before and during pregnancy, respectively (Table 3). The proportions of participants with positive attitudes were significantly higher among female participants (86.7% vs. 67.1%, *p* < 0.001), Saudis (85.6% vs. 74.7%, *p* = 0.015), and married participants (87.2% vs. 67.6% among divorced and 75.0% among widows, *p* < 0.001). Furthermore, positive attitudes were significantly associated with having a low monthly income (88.1% among < 5000 SAR vs. 77.6% among 5000 to 10,000 SAR and 84.1% among > 10,000 SAR, *p* = 0.026) and having no mental disorders (86.9%) or depression (90.0%) compared to those with anxiety (72.9%) or other mental disorders (54.2%). Positive attitudes were significantly lower among participants with 5 to 7 children (76.6%) than those with 1 to 5 children (86.3%) and >7 children (86.7%, Table 4). On the multivariate analysis, positive attitudes were significantly predicted by being a female participant (OR = 2.3, 95% CI, 1.2 to 4.3, *p* = 0.008) and married (OR = 2.2, 95% CI, 1.1 to 4.1, *p* = 0.019), whereas those with other mental disorders were less likely to express positive attitudes (OR = 0.4, 95% CI, 0.1 to 1.0, *p* = 0.045, Table 5).

### 2.4. Practice towards Shaken Baby Syndrome

**Figure 2:**
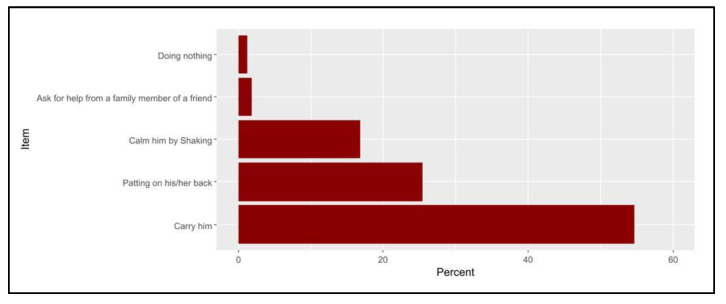
The percentages of the participants’ actions when their baby is crying.

**Figure 3:**
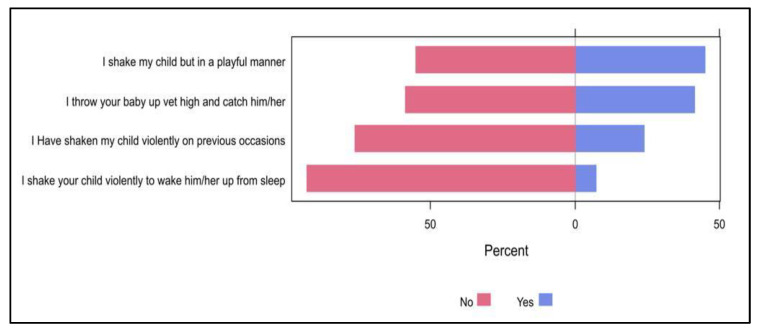
The percentages of participants’ responses regarding their practice towards shaken baby syndrome.

The most frequently reported practices when a baby is crying were carrying the child (54.7%), patting on the child’s back (25.5%), and shaking (16.8%, Figure 2). Less than a half of the participants used to shake their child in a playful manner (44.8%) and throw their baby up high and catch them (41.4%), whereas approximately one-quarter of them (23.9%) had shaken their child violently on previous occasions. Furthermore, only 7.4% of the participants had shaken their child violently to wake them up from sleep (Figure 3).

## 3. Discussion

Shaken baby syndrome (SBS) is an avoidable, severe form of physical child abuse caused by violently shaking an infant by the shoulders, arms, or legs [1].

The present study aimed to assess the level of awareness and knowledge about shaken baby syndrome among parents of the pediatric population in Jeddah, Saudi Arabia.

Previous studies revealed a low level of knowledge among Saudi parents.

Of the study participants, 30.7% said they were aware of shaken baby syndrome (SBS). A total of 32.0% of Saudi respondents to a recent study conducted in 2022 had heard of SBS [14]. According to a 2018 Tabuk survey, 67.39% of participant parents were unaware of the dangers of SBS [18]. These findings report a low level of awareness could be due to the fact there is no enough educational programs targeting parents about such topic.

According to a prior survey conducted in Egypt in 2020, the majority of mothers (80%) did not learn anything about SBS, while only 20% of mothers did [20]. In the same context, Mann et al. (2015) discovered that 54% of individuals were unaware of SBS [21]. Additionally, more than the (57%) of the participants, according to Marcinkowska et al. (2016) [22], have heard of SBS. The difference between two countries explains the importance of having educational programs.

Education of the parents may help to prevent inappropriate treatment of crying newborns, including shaking babies, according to a 2015 Irish study that found that 50% of participants were unaware of shaken baby syndrome [21]. Finally, 40% of participants in a 2019 German study [15] had no prior knowledge of abusive head trauma (AHT) or shaken baby syndrome. The lack of educational initiatives and public awareness campaigns aimed at parents may be the cause of the low level of knowledge found in the current study.

These findings showed that mothers have a substantial lack of knowledge regarding SBS. Given that moms actively participate in SBS prevention, it is an important consideration when developing mother’s SBS preventive programs. According to the “Shaken Baby Syndrome National Center” initiative, non-accidental head injuries dropped by 47% over a three-year period in New York with the provision of SBS information to all parents of newborn children in the hospital (CDC, 2013 [23]. Due to these programs, it was found that knowledge of shaken baby syndrome was considerably higher in women than in men.

In this survey, it was found that among knowledgeable participants, the Internet was the most common information source (46.1%), with social media, family, and neighbors accounting for the majority of sources for 32.3% of them. This result is predictable because of the Internet. This result is to be expected given that the Internet and social media are the main sources of information for many aspects of life. Statistics show that almost 98% of the Saudi Arabian population uses the Internet, and WhatsApp is the most popular social networking in KSA with 30.67 million users [20].

In just 4% of mothers in an Egyptian study, the health care professional was the source of information regarding SBS [20]. Less than 1% of participants found out about SBS through a healthcare provider, according to Mann et al.’s 2015 research [21]. The media was the most prevalent source of information for those who had heard about SBS. Physicians play a major role in informing parents and increasing awareness about SBS during baby visits, vaccination, and primary health care.

Based on the respondents’ sociodemographic traits, the current study indicated no statistically significant disparities in knowledge levels. The knowledge level, on the other hand, was found to be substantially correlated with gender, marital status, and occupation in a prior Saudi study [18].

In previous studies conducted by Berthold et al. [15], Bechtel et al. [24], and Simonnet et al. [25], a substantial link between education level and understanding of SBS was also discovered by Bechtel et al. [24], which was absent from the current investigation. A prior study [26] found a correlation between low socioeconomic position and low educational attainment and all forms of child maltreatment.

The majority of the participants in the current study had a positive attitude (84.0%), and they wished to learn more about SBS. Additionally, before and during pregnancy, 40.1% and 34.3% of respondents desired information on SBS, respectively. Being female, Saudi, married, having a low monthly income, and not having any mental illnesses were all substantially linked to having a positive attitude.

In a Saudi survey conducted in 2022, it was discovered that 82.5% of participants had an optimistic attitude [18] and participants were found to have a high level of positivity and the same low level of knowledge as our study [18]. One of the most crucial aspects of newborn care is knowing how to respond to a crying baby [27]. The health of the infant is negatively impacted by mothers’ incorrect traditional beliefs, knowledge, and practices as well as by their ignorance of infant care [28].

In the postpartum phase, a baby’s persistent crying can leave the parents exhausted, sleep deprived, and irritable [29]. The infant’s uncontrollable sobbing scares the parent because they don’t know why the baby is crying or what to do about it. This exacerbates anger and serves as the primary catalyst for violence. Loss of self-control results from growing rage. Lack of social support might cause such stress to rapidly rise. Parents may become more aggressive and exhibit stronger negative emotional and physiological reactions [30]. In addition, postpartum depression was found to be independent risk factor for aggressive behaviors toward the infant such as shaking regardless of the perceived amount of crying [8,9].

Carrying the kid, patting the child’s back, and shaking were the behaviors associated with baby crying that were most frequently reported in the current study. Less than half of parents used to playfully shake their child or toss and catch their youngster in the air, while 7.4% of parents aggressively shook their child to wake them up from sleep and 23.9% had previously shaken their child violently.

According to research conducted in the United Arab Emirates, 99.1% of mothers nursed their crying infants, 96.9% held them in their arms, 64.7% offered them herbal tea, and 42.2% rocked the infant [31]. According to a different study, 87% of mothers held their infants in their arms, 82% nursed them, and 67% rocked them to sleep during the first 16 weeks of life [32].

The baby may have shaken baby syndrome (SBS) if the parent can’t control their emotions and violently shakes the baby back and forth while holding them around the arms or torso [33]. It has been discovered that as a baby cries more frequently, SBS occurs more frequently as well. Therefore, crying is considered as a trigger mechanism for the SBS [29].

Every professional who works with children and families, as well as every person taking care of a baby or young kid, are also responsible for SBS prevention [34]. The nurse can mitigate the irreparable effects of SBS and assist caregivers in dealing with a screaming infant [35]. The transmission of a strong, concise message by nurses to parents, especially mothers, at multiple times during pregnancy, childbirth, and follow-up doctor visits is the main component of SBS prevention. The nurse has a duty to inform parents, caregivers, medical professionals, and the general public about the dangers of shaking, the commonality of infant crying, a variety of soothing and calming techniques for mothers and children, and how to safely lessen the burden of caring for young children as well as additional support services [36].

## 4. Limitations

Different factors may be considered as limitations in this study. The recruitment method had a potential for ascertainment bias, which limit the generalization of the findings. Using an online survey had the potential for self-selection of the participants among individuals who have a better propensity toward the sharing of personal information and conditions, such as those who are familiar with the use of new media. Furthermore, a potential social desirability bias could be predicted because of the possible imbalance between the very low share of people who have a good understanding of this topic and the very high share of people who want to improve their knowledge. Recall bias is considered as a limitation in self-reporting questionnaires.

## 5. Conclusions

This study found that 30.7% of the participants knew about SBS and the most common sources of knowledge among them were the Internet and social media. Only 32.3% had a good knowledge level with no significant association between knowledge levels and participants’ sociodemographic characteristics. Of them, 84% wanted to know about SBS and 40.1% and 34.3% wanted to know SBS-related information before and during pregnancy, respectively. The positive attitude was significantly higher among female participants, Saudis, married participants, those with low monthly income, and those that have no mental disorders or depression. The most common practices followed when a baby is crying were carrying the child, patting on the child’s back, and shaking. Of them, 41.4% throw their baby up high and catch them and 23.9% shake their child violently. There is a low level of knowledge regarding SBS among the participants in the study. Implementing preventative initiatives is essential and stopping abuse before it starts is prioritized as an approach. Health education programs on SBS for mothers should be conducted throughout the prenatal period.

## Figures and Tables

**Table 1:** Sociodemographic characteristics (N = 524).

Parameter	Category	N (%)
Gender	Male	73 (13.9%)
	Female	451 (86.1%)
Age (years)	<20	14 (2.7%)
	20 to 29	142 (27.1%)
	30 to 39	152 (29.0%)
	40 or more	216 (41.2%)
Nationality	Saudi	445 (84.9%)
	Non-Saudi	79 (15.1%)
Marital status	Married	429 (81.9%)
	Divorced	71 (13.5%)
	Widowed	24 (4.6%)
Educational level	High school or less	101 (19.3%)
	Diploma	53 (10.1%)
	Bachelor’s	315 (60.1%)
	Postgraduate	55 (10.5%)
Occupation	Employee	260 (49.6%)
	Non-employee	76 (14.5%)
	Housewife	188 (35.9%)
Monthly income (SAR/USD)	<SAR 5000 (USD 1350)	226 (43.1%)
	SAR 5000 to 10,000 (USD 1350–2700)	147 (28.1%)
	>SAR 10,000 (>USD 2700)	151 (28.8%)
Number of children	1 to 5	366 (69.8%)
	5 to 7	128 (24.4%)
	>7	30 (5.7%)
Have a chronically ill child at home	Yes	56 (10.7%)
Have any mental disorders	None	421 (80.3%)
	Anxiety	59 (11.3%)
	Depression	20 (3.8%)
	Others	24 (4.6%)

**Table 2:** Factors associated with knowledge levels among participants who were knowledgeable about shaken baby syndrome (N = 161).

Parameter	Category	Knowledge Level		
		Poor, N = 109	Good, N = 52	*p*-Value
Gender	Male	15 (75.0%)	5 (25.0%)	0.456
	Female	94 (66.7%)	47 (33.3%)	
Age (years)	<20	2 (66.7%)	1 (33.3%)	0.762
	20 to 29	25 (61.0%)	16 (39.0%)	
	30 to 39	39 (69.6%)	17 (30.4%)	
	40 or more	43 (70.5%)	18 (29.5%)	
Nationality	Saudi	91 (67.9%)	43 (32.1%)	0.900
	Non-Saudi	18 (66.7%)	9 (33.3%)	
Marital status	Married	86 (65.2%)	46 (34.8%)	0.155
	Divorced	16 (72.7%)	6 (27.3%)	
	Widowed	7 (100.0%)	0 (0.0%)	
Educational level	High school or less	23 (74.2%)	8 (25.8%)	0.134
	Diploma	18 (85.7%)	3 (14.3%)	
	Bachelor’s	58 (63.7%)	33 (36.3%)	
	Postgraduate	10 (55.6%)	8 (44.4%)	
Occupation	Employee	53 (61.6%)	33 (38.4%)	0.172
	Non-employee	13 (81.2%)	3 (18.8%)	
	Housewife	43 (72.9%)	16 (27.1%)	
Monthly income (SAR/USD)	<SAR 5000 (USD 1350)	46 (73.0%)	17 (27.0%)	0.371
	SAR 5000 to 10,000 (USD 1350–2700)	34 (68.0%)	16 (32.0%)	
	>SAR 10,000 (>USD 2700)	29 (60.4%)	19 (39.6%)	
Number of children	1 to 5	72 (63.7%)	41 (36.3%)	0.139
	5 to 7	34 (79.1%)	9 (20.9%)	
	>7	3 (60.0%)	2 (40.0%)	
	Have a chronically ill child at home	18 (75.0%)	6 (25.0%)	
Have any mental disorders	None	84 (65.1%)	45 (34.9%)	0.347
	Anxiety	15 (75.0%)	5 (25.0%)	
	Depression	4 (66.7%)	2 (33.3%)	
	Others	6 (100.0%)	0 (0.0%)	

**Table 3:** Description of participants’ attitudes towards shaken baby syndrome (N = 524).

Parameter	Category	N (%)
Want to know more about shaken baby syndrome	No	84 (16.0%)
	Yes	440 (84.0%)
If yes, from which source?	A doctor or medical staff during the vaccination period	120 (28.6%)
	Internet and social media	156 (37.1%)
	Awareness campaign	103 (24.5%)
	Medical books and bulletins	41 (9.8%)
	Missing	20 (4.5%)
When is the preferred time period that you want to obtain information about shaken baby syndrome ^¥^	Before pregnancy	172 (40.1%)
	During pregnancy	147 (34.3%)
	A week after giving birth	33 (7.7%)
	During vaccination visits to the child	77 (17.9%)
	Missing	11 (2.5%)

^¥^ the variable is based on 440 participants who wanted to know about shaken baby syndrome.

**Table 4:** Factors associated with participants’ attitudes about shaken baby syndrome (N = 524).

Parameter	Category	Attitude		
		Negative, N = 84	Positive, N = 440	*p*-Value
Gender	Male	24 (32.9%)	49 (67.1%)	<0.001
	Female	60 (13.3%)	391 (86.7%)	
Age (years)	<20	1 (7.1%)	13 (92.9%)	0.502
	20 to 29	21 (14.8%)	121 (85.2%)	
	30 to 39	30 (19.7%)	122 (80.3%)	
	40 or more	32 (14.8%)	184 (85.2%)	
Nationality	Saudi	64 (14.4%)	381 (85.6%)	0.015
	Non-Saudi	20 (25.3%)	59 (74.7%)	
Marital status	Married	55 (12.8%)	374 (87.2%)	<0.001
	Divorced	23 (32.4%)	48 (67.6%)	
	Widow	6 (25.0%)	18 (75.0%)	
Educational level	High school or less	14 (13.9%)	87 (86.1%)	0.084
	Diploma	15 (28.3%)	38 (71.7%)	
	Bachelors	47 (14.9%)	268 (85.1%)	
	Postgraduate	8 (14.5%)	47 (85.5%)	
Occupation	Employee	42 (16.2%)	218 (83.8%)	0.776
	Non-employee	14 (18.4%)	62 (81.6%)	
	Housewife	28 (14.9%)	160 (85.1%)	
Monthly income (SAR)	<5000 (SAR 1350)	27 (11.9%)	199 (88.1%)	0.026
	5000 to 10,000 (SAR 1350–2700)	33 (22.4%)	114 (77.6%)	
	>10,000 (>SAR 2700)	24 (15.9%)	127 (84.1%)	
Number of children	1 to 5	50 (13.7%)	316 (86.3%)	0.036
	5 to 7	30 (23.4%)	98 (76.6%)	
	>7	4 (13.3%)	26 (86.7%)	
Have a chronically ill child at home	No	75 (16.0%)	393 (84.0%)	0.993
	Yes	9 (16.1%)	47 (83.9%)	
Have any mental disorders	None	55 (13.1%)	366 (86.9%)	<0.001
	Anxiety	16 (27.1%)	43 (72.9%)	
	Depression	2 (10.0%)	18 (90.0%)	
	Others	11 (45.8%)	13 (54.2%)	

**Table 5:** Results of the regression analysis for the predictors of positive attitudes towards SBS.

Parameter	Category	OR	95% CI	*p*-Value
Gender	Male	—	—	
	Female	2.32	1.23, 4.31	0.008
Nationality	Saudi	—	—	
	Non-Saudi	0.78	0.40, 1.59	0.485
Marital status	Divorced	—	—	
	Married	2.18	1.11, 4.14	0.019
	Widow	1.52	0.50, 5.15	0.478
Monthly income (SAR)	<5000	—	—	
	5000 to 10,000	0.58	0.32, 1.06	0.075
	>10,000	0.81	0.42, 1.58	0.536
Number of children	1 to 5	—	—	
	5 to 7	0.62	0.35, 1.10	0.099
	>7	1.86	0.62, 7.14	0.308
Have any mental disorders	None	—	—	
	Anxiety	0.54	0.27, 1.13	0.090
	Depression	1.87	0.46, 12.9	0.444
	Others	0.37	0.14, 1.00	0.045

## Data Availability

Data available on request due to ethical restriction.

## References

[1] Christian C.W., Block R. (2009). Abusive head trauma in infants and children. Pediatrics.

[2] Stewart T.C., Polgar D., Gilliland J., Tanner D.A., Girotti M.J., Parry N., Fraser D.D. (2011). Shaken baby syndrome and a triple dose strategy for its prevention. J. Trauma.

[3] Ludwig S., Warman M. (1984). Shaken baby syndrome: A review of 20 cases. Ann. Emerg. Med..

[4] Lopes N.R., Eisenstein E., Williams L.C. (2013). Abusive head trauma in children: A literature review. J. Pediatr..

[5] Minns R.A., Jones P.A., Mok J.Y.Q. (2008). Incidence and demography of non-accidental head injury in southeast Scotland from a national database. Am. J. Prev. Med..

[6] Kelly P., Farrant B. (2008). Shaken baby syndrome in New Zealand, 2000–2002. J. Paediatr. Child Health.

[7] Gilbert R., Kemp A., Thoburn J., Sidebotham P., Radford L., Glaser D., MacMillan H.L. (2009). Recognising and responding to child maltreatment. Lancet.

[8] Fujiwara T., Yamaoka Y., Morisaki N. (2016). Self-Reported Prevalence and Risk Factors for Shaking and Smothering Among Mothers of 4-Month-Old Infants in Japan. J. Epidemiol..

[9] Adamsbaum C., Grabar S., Mejean N., Rey-Salmon C. (2010). Abusive head trauma: Judicial admissions highlight violent and repetitive shaking. Pediatrics.

[10] Radesky J.S., Zuckerman B., Silverstein M., Rivara F.P., Barr M., Taylor J.A., Lengua L.J., Barr R.G. (2013). Inconsolable infant crying and maternal postpartum depressive symptoms. Pediatrics.

[11] Miehl N.J. (2005). Shaken baby syndrome. J. Forensic Nurs..

[12] Hoffman J.M. (2005). A case of shaken baby syndrome after discharge from the newborn intensive care unit. Adv. Neonatal Care.

[13] Black D.A., Heyman R.E., Slep A.M.S. (2001). Risk factors for child physical abuse. Aggress. Violent Behav..

[14] National Center for Injury Prevention and Control (USA), Centers for Disease Control and Prevention (USA) (2010). A Journalist’s Guide to Shaken Baby Syndrome: A Preventable Tragedy Centers for Disease Control and Prevention. www.cdc.gov.

[15] Berthold O., Clemens V., Witt A., Brähler E., Plener P.L., Fegert J.M. (2019). Awareness of abusive head trauma in a German population-based sample: Implications for prevention. Pediatr. Res..

[16] Foley S., Kovács Z., Rose J., Lamb R., Tolliday F., Simons-Coghill M., Stephens A., Scheiber D., Toma A., Asbóth K. (2013). International collaboration on prevention of shaken baby syndrome–an ongoing project/intervention. Paediatr. Int. Child Health.

[17] Alshahrani A.N., Alshahrani M.N., Ahmed A.B. (2018). Evaluation of knowledge regarding shaken baby syndrome among parents in Tabuk City. Egypt. J. Hosp. Med..

[18] Alomran H.I., Alkharaan Z.I., Aldawsari K.M., Aldakkan O.Z., Alatif H.M., Mohamed M.Z.E. (2022). Parental awareness, knowledge, and attitude about shaken baby syndrome in Riyadh, Saudi Arabia: A cross-sectional study. Pan Afr. Med. J..

[19] (2001). American Academy of Pediatrics Committee on Child Abuse and Neglect Shaken baby syndrome: Rotational cranial injuries-technical report. Pediatrics.

[20] El Sayed A.I., Mohamed S.A. (2020). Effect of Educational Materials on Mother’s Awareness, Knowledge and Behavior Regarding the Dangers of Shaken Baby Syndrome. IOSR J. Nurs. Health Sci..

[21] Mann A.K., Rai B., Sharif F., Vavasseur C. (2015). Assessment of parental awareness of the shaken baby syndrome in Ireland. Eur. J. Pediatr..

[22] Marcinkowska U., Tyrała K., Paniczek M., Ledwoń M., Jośko-Ochojska J. (2021). Evaluation of knowledge regarding shaken baby syndrome among parents and medical staff. Minerva Pediatr..

[23] Centers for Disease Control and Prevention, Prevention, National Center for Injury Prevention and Control (2013). Preventing Shaken Baby Syndrome: A Guide for Health Departments and Community-Based Organizations.

[24] Bechtel K., Le K., Martin K., Shah N., Leventhal J., Colson E. (2011). Impact of an educational intervention on caregivers’ beliefs about infant crying and knowledge of shaken baby syndrome. Acad. Pediatr..

[25] Simonnet H., Laurent-Vannier A., Yuan W., Hully M., Valimahomed S., Bourennane M., Chevignard M. (2014). Parents’ behavior in response to infant crying: Abusive head trauma education. Child Abus. Negl..

[26] Kelly P., Thompson J., Koh J., Ameratunga S., Jelleyman T., Percival T., Elder H., Mitchell E.A. (2017). Perinatal risk and protective factors for pediatric abusive head trauma: A multicenter case-control study. J. Pediatr..

[27] Yildiz D. (2008). Postpartum counseling needs and approaches of mothers regarding baby care. Gulhane Tip Jour.

[28] Koc F., Aksit S., Turhan T., Ersahin Y., Tomba A., Halicioglu O., Aslan A., Koturoglu G., Aydin C., Cetin S. (2012). Shaken baby syndrome: Case report. Turk. Klin. J. Med. Sci..

[29] Sahin F., Tasar M.A. (2012). Shaken baby syndrome and prevention programme. Turk. Pediatry Arch..

[30] Crouch J.L., Hiraoka R., McCanne T.R., Reo G., Wagner M.F., Krauss A., Milner J.S., Skowronski J.J. (2018). Heart Rate and Heart Rate Variability in Parents at Risk for Child Physical Abuse. J. Interpers. Violence.

[31] Abdulrazzaq Y.M., Al Kendi A., Nagelkerke N. (2009). Soothing methods used to calm a baby in an Arab country. Acta Paediatr..

[32] Howard C.R., Lanphear N., Lanphear B.P., Eberly S., Lawrence R.A. (2006). Parental responses to infant crying and colic: The effect on breastfeeding duration. Breastfeed. Med..

[33] Cansever Z., Tasar M.A., Sahin F., Camurdan A.D., Beyazova U. (2012). Knowledge and attitudes of families about shaken baby syndrome. Gazi Med. J..

[34] Susamma T. (2016). Soothing Crying Babies and Preventing Shaken Baby Syndrome. Int. J. Nurs. Educ..

[35] Lekarski P., Talarowska M., Florkowski A., Mossakowska J., Gałecki P. (2010). The shaken baby syndrome as a kind of domestic abuse. Polski Merkur. Lek. Organ Polskiego Towar. Lek.

[36] Taşar M.A., Şahin F., Polat S., İlhan M., Çamurdan A., Dallar Y., Beyazova U. (2014). Long-term outcomes of the shaken baby syndrome prevention program: Turkey’s experience. Turk. Arch. Pediatr..

